# Long-term Adherence to Healthy Dietary Guidelines and Chronic Inflammation in the Prospective Whitehall II Study^☆^

**DOI:** 10.1016/j.amjmed.2014.10.002

**Published:** 2015-02

**Authors:** Tasnime N. Akbaraly, Martin J. Shipley, Jane E. Ferrie, Marianna Virtanen, Gordon Lowe, Mark Hamer, Mika Kivimaki

**Affiliations:** a Inserm U710, Montpellier, F-34000, France; b University Montpellier II, Montpellier, France; EPHE, Paris, France; c Department of Epidemiology and Public Health, University College London, London, United Kingdom; d School of Social and Community Medicine, University of Bristol, Bristol, United Kingdom; e Finnish Institute of Occupational Health, Helsinki, Finland; f Institute of Cardiovascular and Medical Sciences, University of Glasgow, Glasgow, United Kingdom

**Keywords:** Alternative Healthy Eating Index, Diet quality indices, Inflammatory marker, Interleukin-6, Middle-aged population, Nutritional Epidemiology, Prospective cohort

## Abstract

**Background:**

Inflammation plays an important role in the cause of cardiovascular diseases and may contribute to the association linking an unhealthy diet to chronic age-related diseases. However, to date the long-term associations between diet and inflammation have been poorly described. Our aim was to assess the extent to which adherence to a healthy diet and dietary improvements over a 6-year exposure period prevented subsequent chronic inflammation over a 5-year follow-up in a large British population of men and women.

**Methods:**

Data were drawn from 4600 adults (mean ± standard deviation, age 49.6 ± 6.1 years, 28% were women) from the prospective Whitehall cohort II study. Adherence to a healthy diet was measured using Alternative Healthy Eating Index (AHEI) scores in 1991-1993 (50.7 ± 11.9 points) and 1997-1999 (51.6 ± 12.4 points). Chronic inflammation, defined as average levels of serum interleukin-6 from 2 measures 5 years apart, was assessed in 1997-1999 and 2002-2004.

**Results:**

After adjustment for sociodemographic factors, health behaviors, and health status, participants who maintained a high AHEI score (ie, a healthy diet, n = 1736, 37.7%) and those who improved this score over time (n = 681, 14.8%) showed significantly lower mean levels of interleukin-6 (1.84 pg/mL, 95% confidence interval [CI], 1.71-1.98 and 1.84 pg/mL, 95% CI, 1.70-1.99, respectively) than those who had a low AHEI score (n = 1594, 34.6%) over the 6-year exposure period (2.01 pg/mL, 95% CI, 1.87-2.17).

**Conclusions:**

These data suggest that maintaining and improving adherence to healthy dietary recommendations may reduce the risk of long-term inflammation.

**Clinical Significance**

• Data from British adults suggest that long-term good and improving adherence to healthy recommendations provided by the Alternative Healthy Eating Index score are associated with lower levels of chronic inflammation assessed by serum interleukin 6.
• Adverse dietary effects on inflammatory markers were observed when adherence to healthy dietary recommendations was poor in the repeated measurements over a 6-year period.

The impact of an unhealthy diet on the risk of specific age-related diseases, such as cardiovascular disease^1-6^ and type 2 diabetes,^7,8^ has been shown in various independent studies. Inflammatory processes have been suggested partly to underlie these associations.^9^ In agreement with this hypothesis, a recent study applying Mendelian randomization provided convincing evidence for a causal role of an inflammatory marker interleukin (IL)-6 in coronary heart disease.^10^ However, the extent to which modifiable factors, such as a healthy diet, might protect against systemic chronic inflammation remains unclear.

Many observational studies on the overall diet–inflammation association have been based on cross-sectional data^11^ that cannot separate the impact of diet on inflammation from possible reverse causation effects (ie, the impact of chronic inflammation on dietary habits). Of the recent cohort studies with a prospective design, one reported that dietary pattern reflecting a high n-6:n-3 fatty acid intake ratio was associated with higher serum levels of C-reactive protein (CRP) measured 13 years later.^12^ Another longitudinal study showed that long-term adherence to the Alternative Healthy Eating Index (AHEI), a multicomponent measure of healthy diet, was associated with CRP but not with IL-6 serum levels.^13^ A major limitation in all these cross-sectional and longitudinal studies is the lack of repeat data on inflammation because the assessment of inflammation at one point in time only precludes distinctions between short-term (acute) and longer-term (chronic) inflammatory processes.

Several randomized controlled trials have been performed to determine the association between diet and inflammatory markers,^9,14-23^ most of them assessing the beneficial impact of the Mediterranean diet.^14,16-22^ Results from these trials have shown a positive impact of short-term adherence to healthy dietary guidelines on inflammation^15-23^ in obese participants,^17,19,21^ participants with type 2 diabetes,^9,15^ participants with a high risk of cardiovascular disease,^18^ participants with the metabolic syndrome,^16^ and participants with prevalent myocardial infarction.^22^ On the other hand, when intervention studies have been performed in nonclinical and healthier populations,^14,23^ no association with CRP or IL-6 serum levels has been observed. However, none of these studies assessed whether long-term adherence to a healthy diet or improvements in diet were associated with subsequent chronic inflammation in a general population.

To address some of these limitations in the current evidence, we analyzed data from a large British occupational cohort, the Whitehall II study, in which adherence to the dietary guidelines provided by the AHEI has been shown to reduce the risk of cardiovascular death^24^ and increase the likelihood of reversion of the metabolic syndrome in participants with central obesity and high triglyceride levels.^25^ The aim of the present article is to examine associations of the AHEI score and change in AHEI score over a 6-year exposure period with subsequent chronic inflammation, assessed by average serum levels of IL-6 from 2 measures 5 years apart.

## Materials and Methods

### Study Population

Participants of the Whitehall II study were London-based office staff, aged 35 to 55 years, who worked in 20 civil service departments at study inception.^26^ Baseline screening (phase 1: 1985-1988, n = 10,308) comprised a clinical examination and a self-administered questionnaire. Subsequent phases of data collection alternated between a clinical examination and a questionnaire survey (phase 3: 1991/1993, n = 8815; phase 5: 1997/1999, n = 7263; phase 7: 2002/2004, n = 6943) and a postal questionnaire alone (phases 2, 4, 6, and 8). Phase 3 is considered the baseline for the purpose of this study because it represents the first assessment of dietary intakes. The current study uses clinical and questionnaire data on overall diet and change in diet between 1991/1993 and 1997/1999, and the average levels of serum IL-6 measured in 1997/1999 and 2002/2004. The University College London Ethics Committee approved the study. After the participants were given a complete description of the study, written informed consent was obtained from all participants.

### Data Collection

#### Diet Quality Using the Alternative Healthy Eating Index in 1991/1993 and 1997/1999

At both examinations, dietary intakes were assessed using a semiquantitative food-frequency questionnaire with 127 food items, as described previously.^24,27^ The AHEI^5^ was scored on the basis of the intake levels of 9 components (Appendix Table 1, available online): (1) vegetables, (2) fruits, (3) nuts and soy, (4) ratio of white meat (seafood and poultry) to red meat, (5) trans fat, (6) total fiber, (7) ratio of polyunsaturated fat to saturated fat, (8) long-term multivitamin use, and (9) alcohol consumption. Higher scores corresponded to a healthier diet.

#### Inflammatory Markers Assessment in 1997/1999 and 2002/2004

IL-6, a marker of systemic inflammation, was measured using a high-sensitivity enzyme-linked immunosorbent assay (R&D Systems, Oxford, UK).^28,29^ Procedures are detailed in the Appendix, available online. Long-term inflammation was assessed as the average of 2 IL-6 measures in 1997/1999 and 2002/2004 (n = 3632) or by a single measure if only 1 was available (n = 968). CRP was also measured using a high-sensitivity immunonephelometric assay in a BN ProSpec nephelometer (Dade Behring, Milton Keynes, UK),^28,29^

#### Covariates Assessed at Baseline

Sociodemographic variables included sex, age, ethnicity (white/South Asian/black), living alone (yes/no), and socioeconomic status (low/intermediate/high). Health behaviors were smoking habits (never/former/current), total energy intake (kilocalories per day), and physical activity (inactive/moderately active/active).^30^ Health status covariates included depressive symptoms assessed using the 4-item depression subscale of the 30-item General Health Questionnaire^31^; prevalent coronary heart disease (denoted by clinically verified nonfatal myocardial infarction or definite angina); hypertension (defined by systolic/diastolic blood pressure ≥140/90 mm Hg, respectively, or use of antihypertensive drugs); type 2 diabetes (diagnosed according to the World Health Organization definition); serum high-density lipoprotein cholesterol measured in millimoles/liter; body mass index (weight in kilograms/height in meters squared); and self-reported use of anti-inflammatory medication (eg, nonsteroidal anti-inflammatory drugs and corticosteroids).

### Statistical Analysis

The distribution of average IL-6 assessed in 1997/1999 or 2002/2004 was normalized by logarithmic transformation. The overall AHEI score and its component scores in 1991/1993 were dichotomized as high and low according to the median value (detailed in Appendix Table 1, available online).

To analyze sustained adherence to a healthy diet and improvements in diet, AHEI scores in 1991/1993 and 1997/1999 were categorized as high or low according to the median AHEI score in 1991/1993.^32^ Thus, 4 categories of change in AHEI score over a 6-year period were defined: participants who maintained a high score (1991/1993 and 1997/1999 scores ≥51.5), participants who maintained a low score (1991/1993 and 1997/1999 scores <51.5), participants whose AHEI score improved (1991/1993 score <51.5 and 1997/1999 score ≥51.5), and participants whose AHEI score deteriorated (1991/1993 score ≥51.5 and 1997/1999 score <51.5 points).

Regression models were used to calculate adjusted geometric mean levels of IL-6 for each AHEI score category and the percentage difference (calculated as exp[regression coefficient] – 1)*100) in average levels associated with change in AHEI score. Models were first adjusted for age, sex, socioeconomic status, ethnic group, and use of anti-inflammatory drugs (model 1), and further adjusted for other covariates (model 2), including living alone, smoking status, physical activity, total energy intake, coronary heart disease, hypertension, diabetes, body mass index, and high-density lipoprotein cholesterol (as defined in “Covariates Assessed at Baseline” section).

In subsidiary analyses, we used linear regression to assess the reverse association: whether IL-6 level assessed in 1991/1993 was associated with 6-year change in AHEI score between 1991/1993 and 1997/1999 and the 11-year change in AHEI score between 1991/1993 and 2002/2004. All analyses were conducted using SAS software, version 9 (SAS Institute Inc, Cary, NC).

## Results

### Participant Characteristics

**Figure 1:**
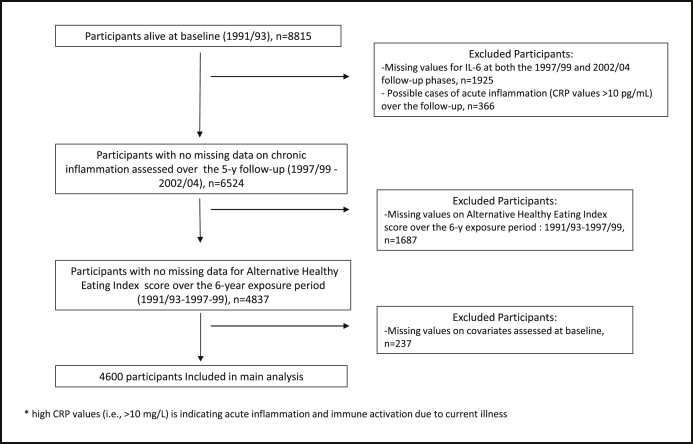
Flow chart mapping the selection of the 4600 Whitehall II participants included in the present analyses.

The present analyses were restricted to participants with complete data on inflammation, change in diet, and covariates (n = 4600). Possible cases of acute inflammation and immune activation due to current illness (defined as having CRP values >10 pg/mL) were excluded because these may reflect short-term responses not representative of usual levels of chronic low-grade inflammation. A flow chart depicting participant selection is shown in Figure 1.

Compared with participants excluded from the present analyses, those included were more likely to be men and younger, with higher socioeconomic status and healthy behaviors, and were less likely to have coronary heart diseases or cardiometabolic disorders. Lower average mean IL-6 values were observed in those included (1.63 pg/mL, 95% confidence interval [CI], 1.60-1.66) compared with those excluded (2.10 pg/mL; 95% CI, 2.03-2.14; *P* < .001). In regard to the exposure, the AHEI score in 1991/1993 was higher in those included compared with those excluded (50.7 ± 11.9 points vs 49.4 ± 12.7 points, *P* < .001); however, no statistically significant difference was observed for the AHEI score in 1997/1999 or for the 6-year change in the AHEI score.

In the 4600 participants included in the present analyses, the mean (± standard deviation) score of AHEI was 50.7 ± 11.9 points in 1991/1993 and 51.6 ± 12.4 points in 1997/1999. The Pearson correlation coefficient between AHEI scores at 1991/1993 and 1997/1999 was 0.62 (95% CI, 0.60-0.64). Table 1 shows the mean long-term IL-6 over the 5-year follow-up (1997/1999 and 2002/2004) by characteristics of the 4600 participants. Except for depressive symptoms, all other socioeconomic, heath behavior, and health status factors were associated with average levels of IL-6 and were then considered as covariates in further multivariable adjusted analyses.

### AHEI Score at Baseline (1991/93) and Average Levels of IL-6 over the 5-Year Follow-up (1997/99-2002/04)

**Appendix Figure 1:**
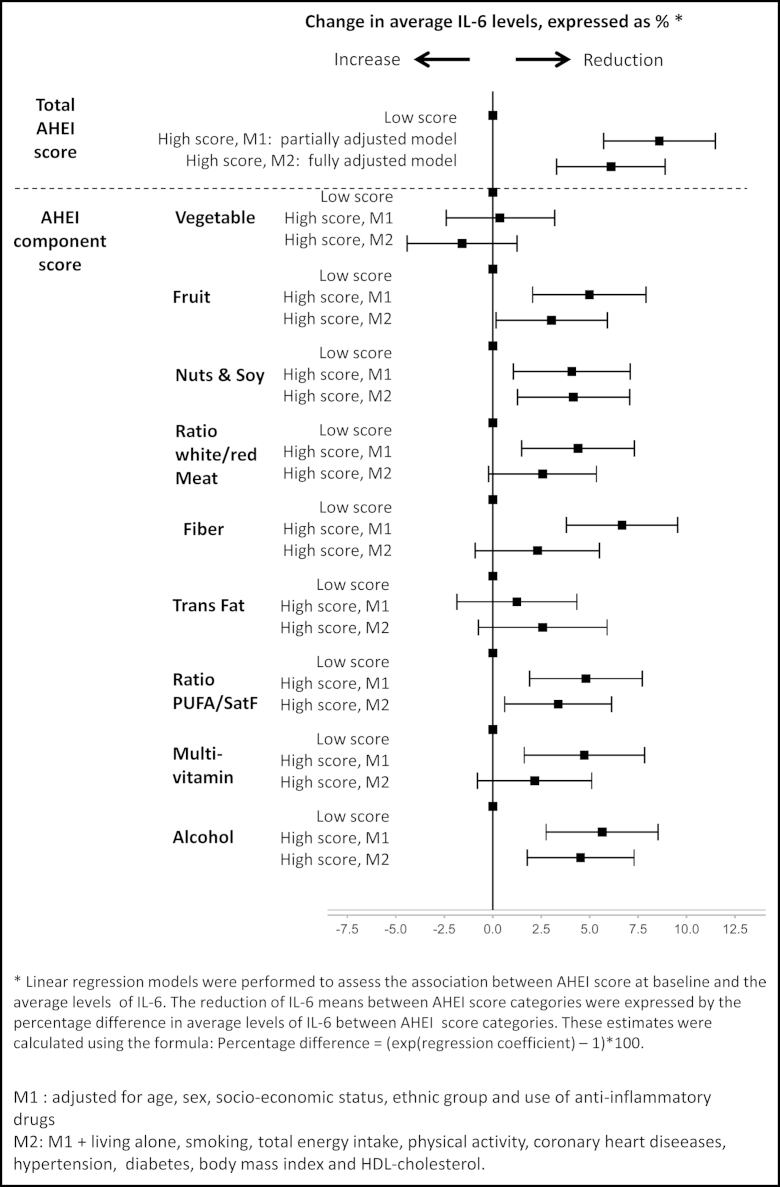
Difference in average interleukin-6 levels (1997/1999 and 2002/2004) as a function of high versus low overall AHEI score and AHEI component scores at baseline (1991/1993) in 4600 participants from the Whitehall II cohort. AHEI = Alternative Healthy Eating Index; IL = interleukin; PUFA = polyunsaturated fatty acid; SatF = saturated fat.

Compared with having a low AHEI score at baseline (below median value of 51.5 points, n = 2275), a high AHEI score (median or greater, n = 2325) was associated with lower average levels of IL-6 (regression coefficient, −0.087, *P* < .001 in model 1 adjusted for age, sex, ethnicity, socioeconomic status, and use of anti-inflammatory drugs and −0.066, *P* < .001 in model 2 further adjusted for health behaviors and health status factors). All the AHEI components, with the exception of vegetable and trans-fat components, were associated with subsequent average levels of IL-6 in model 1. In model 2, only high scores in the fruit, nuts and soy, ratio of polyunsaturated to saturated fat, and moderate alcohol intake remained significantly associated with lower levels of chronic inflammation (Appendix Figure 1, available online).

### Change in AHEI over the 6-Year Exposure Period (1991/93-1997/99) and IL-6 levels over the 5-Year Follow-up (1997/99-2002/04)

**Figure 2:**
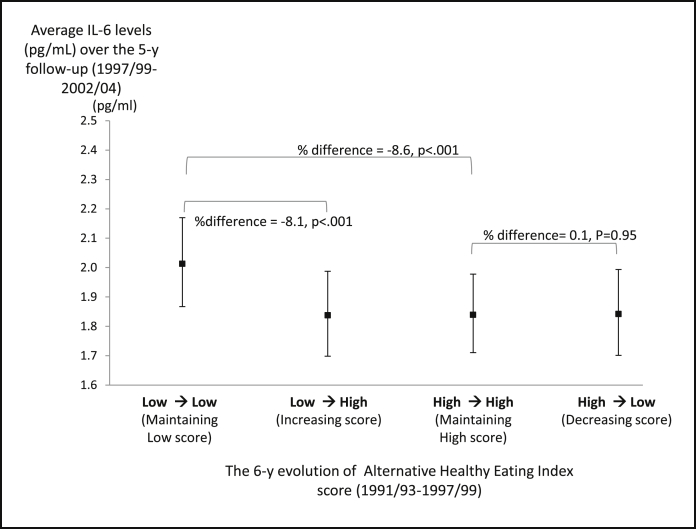
Average levels of IL-6 over 2 measures 5 years apart according to 6-year changes in the AHEI score among 4600 participants from the Whitehall II cohort. Adjusted geometric mean levels of IL-6 were estimated from linear regression models for 4 categories: participants who maintained a low AHEI score over the exposure period, participants who maintained high AHEI score, participants who increased their score, and participants who decreased their score. Models were fully adjusted for sex, age, ethnic group, socioeconomic status, use of anti-inflammatory drugs, living alone, smoking, physical activity, total energy intake, coronary heart diseases, hypertension, diabetes, body mass index, and high-density lipoprotein cholesterol. % difference (exp[regression coefficient] – 1)*100; a negative percentage difference expressed the percentage reduction in IL-6 levels in the relevant exposure group compared with the reference group.

The percentage difference in the average of 2 measures of IL-6 according to the AHEI change categories are shown in Table 2 and Figure 2. Compared with participants who maintained a lower AHEI score (n = 1591, 34.6%) over the 6-year exposure period, those who improved their adherence to AHEI guidelines (n = 681, 14.8%) and those who maintained high AHEI scores (n = 1736, 37.7%) over this period had lower average IL-6 values after adjustment for age, sex, ethnicity, socioeconomic status, and use of anti-inflammatory drugs. Further adjustment slightly attenuated but did not remove these associations (Table 2). Among the 2275 participants who had a lower AHEI score in 1991/1993, each 10-point increase in AHEI score over the 6-year exposure period was associated with a reduction of 4.2% in subsequent average levels of IL-6 (*P* < .001) (results not shown).

As illustrated in Figure 2, there was no significant difference in average IL-6 levels between participants who decreased their AHEI score between 1991/1993 and 1997/1999 (n = 589, 12.8%) and participants who maintained a high AHEI score over the 6-year exposure period, suggesting that the adverse effect of diet on inflammatory markers is detectable only when poor adherence to healthy dietary recommendations is maintained over a long period.

### Sensitivity Analyses

As sensitivity analyses, models were repeated in participants for whom measures of IL-6 were available both in 1997/1999 and 2002/2004 (n = 3632), excluding those with a measure at 1 phase only. Similar results were observed (Table 2). Analyses were also repeated after considering different categories of long-term adherence to AHEI. Adherence to a healthy diet and improvements in diet were built according to quartile values (third quartile or greater, 59.5 vs <59.5 points) of the AHEI score in 1991/1993 (rather than median value). Again, similar trends were obtained (Appendix Table 3, available online).

Furthermore, instead of considering IL-6 measures, we repeated analyses by considering CRP measures; similar associations between change in AHEI score over the 6-year exposure period (1991/1993 to 1997/1999) and average levels of CRP over the 5-year follow-up (1997/1999 to 2002/2004) were observed (Appendix Table 4, available online).

Because IL-6 is involved in physiopathologic processes leading to chronic disease and might modify dietary behaviors, we performed supplementary analyses to assess whether the level of IL-6 assessed in 1991/1993 was related to subsequent 6-year and 11-year change in AHEI score using linear regression models. Although IL-6 levels were cross-sectionally associated with AHEI score at phase 3 (regression coefficient, −0.401; standard error, 0.097; *P* < .001), they were not associated with subsequent change in AHEI score over follow-up (Appendix Table 2, available online), making it less probable that the diet and inflammation association we reported might be due to reverse causality, that is, an effect of inflammation on diet.

## Discussion

The present study examined the association between long-term adherence to dietary guidelines and subsequent chronic inflammation assessed by average levels of IL-6 measured at the beginning and the end of a 5-year follow-up period. The main findings showed that maintaining a healthy diet score or improving adherence to a healthy diet was associated with lower mean levels of IL-6 compared with maintaining a poor dietary score over the 6-year exposure period. This association was independent of socioeconomic factors, health behavior (smoking, physical activity, and total energy intake), and health status (body mass index, use of anti-inflammatory drugs, and cardiometabolic disorders/diseases).

Our analysis of associations between long-term dietary behavior and changes in dietary behavior over time, and subsequent chronic inflammation constitutes a novel approach to the diet and inflammation relationship. In addition to finding that participants who improved or maintained a healthy diet had lower levels of IL-6, we also found that participants whose healthy diet score deteriorated over the 6-year exposure period had subsequent levels of IL-6 similar to those who maintained a high score or improved their score over the same period. This finding suggests that unhealthy dietary behaviors have a deleterious impact on chronic inflammation only when maintained over a long period, a hypothesis that needs to be tested in future studies.

Although previous studies in this field have had methodological limitations, our results are consistent with the diet–inflammation association reported in some previous observational studies.^11^ Studies that have assessed overall diet by extracting dietary patterns using statistical methods, mainly principal component analyses, have shown healthy dietary patterns, generally characterized by higher intake of favorable food groups such as fruits, vegetables, fish, white meat, and whole grains, to be associated with lower levels of CRP^12,33-39^ and IL-6.^33,38,39^ They have also shown that unhealthy dietary patterns— characterized by sweetened desserts, refined grains, processed/red meat, high fat dairy products, and pizzas—to be associated with higher levels of CRP^34,35,37,39,40^ or IL-6.^34,35,40^ We did not observe an association between high overall consumption of vegetables and IL-6. Some previous studies have linked use of cruciferous, but not noncruciferous, vegetables to lower IL-6 levels.^41^

A second set of studies that has assessed food intake using dietary indices^13,42-47^ has led to mixed findings. For example, in studies examining a Mediterranean diet, 2 investigations showed an inverse association between high adherence and levels of both IL-6 and CRP,^43,46^ and one reported a significant association with IL-6 but not CRP.^44^ In regard to the specific dietary index used in the present study, the AHEI, findings from the Nurses’ Health Study have consistently shown an inverse association between AHEI score and levels of CRP and IL-6 in women,^2,13^ although in studies using the Healthy Eating Index (a measure from which the AHEI has been derived), corresponding associations were missing.^42,46^ Likely sources of inconsistency are the cross-sectional design of most existing studies^42-47^ and the assessment of inflammation at one point in time only. By showing that sustained adherence to a healthy diet is robustly associated with subsequent long-term levels of IL-6 assessed twice over a 5-year follow-up, our findings provide strong evidence on the long-term impact of adherence to healthy dietary guidelines on subsequent chronic inflammation in a large population of men and women.

In an additional novel departure, we identified the dietary components most strongly associated with levels of IL-6. After full adjustment, the fruit, nuts and soy, polyunsaturated to saturated fatty acids ratio, and moderate alcohol components were associated with subsequent lower average IL-6 levels. The antioxidants contained in fruits and the high levels of polyunsaturated fat provided by nuts and soy products, which will contribute to a favorable polyunsaturated to saturated fatty acids ratio, may contribute synergistically to reducing proinflammatory stimuli and thus prevent inflammation and the induced secretion of inflammatory cytokines.^48^ The positive impact of moderate alcohol consumption on low-grade inflammation reported in our study is also concordant with the literature, although the underlying mechanisms are still under debate.^49^

### Study Limitations

First, the assessment of dietary intake using a semiquantitative food frequency questionnaire covered only specific foods. This method is recognized to be less precise than dietary assessment by the food diary method. Second, we assessed long-term inflammation using average IL-6 levels assessed twice over 5 years of follow-up. However, having a high IL-6 level at the beginning and end of the follow-up period is not necessarily an indicator of chronic inflammation because repeated short-term inflammatory responses are possible. To address this issue, we used repeat measures of IL-6 and removed participants with acute inflammatory responses (CRP >10) at each measurement. Third, because most of the Whitehall II study participants were office-based civil servants on recruitment to the study, our results may not be generalizable to the wider British population.^26^ Finally, as in every observational study, we cannot exclude that possibility that the diet–inflammation association observed may have been generated by unmeasured confounders, despite the extensive adjustment for a large range of sociodemographic, health behavior, and health status factors.

## Conclusions

Our study is the first to show that sustained adherence and improved adherence to healthy dietary guidelines, as exemplified by the AHEI, are associated with lower levels of subsequent chronic inflammation assessed as serum IL-6 in a white-collar population of men and women. This study reinforces the recommendations of following dietary guidelines that encourage dietary intake similar to what is assessed in the AHEI.

## Figures and Tables

**Appendix Table 1:** Construction of Alternative Healthy Eating Index Scores

Components∗		Criteria for Minimum Scores	Criteria for Maximum Scores	Possible Score Range	AHEI Scores in the Participants in 1991/1993	
					Mean Score (SD)	Median Score
Vegetable (serving/d)		0	5	0-10	5.6 (2.7)	6
Fruit (serving/d)		0	4	0-10	6.1 (3.0)	6
Nuts and soy (serving/d)		0	1	0-10	3.2 (3.0)	3
Ratio of white to red meat		0	4	0-10	5.4 (2.8)	6
Trans fat (% of energy )		≥4	≤0.5	0-10	8.4 (2.8)	10
Total fiber (% of energy)		0	24	0-10	7.8 (3.0)	10
Ratio of polyunsaturated to saturated fat		≤0.1	≥1	0-10	5.2 (2.7)	5
Duration of multivitamin use		<5 y	≥5 y	2.5-7.5	4.2 (2.4)	2.5
Alcohol serving/d	Men	0 or >3.5	1.5-2.5	0-10	4.8 (3.7)†	5†
Alcohol serving/d	Women	0 or >2.5	0.5-1.5	0-10		
Total score				2.5-87.5	50.7 (11.9)	51.5

AHEI = Alternative Healthy Eating Index; SD = standard deviation.

∗ Each AHEI component contributed from 0 to 10 points to the total AHEI score, except the multivitamin component, which was dichotomous and contributing 2.5 points (for nonuse) or 7.5 points (for use). A score of 10 indicates that the recommendations were fully met, whereas a score of 0 represents the least healthy dietary behavior. Intermediate intakes were scored proportionately between 0 and 10.

† Median score for men and women combined.

**Appendix Table 2:** Association between Change∗ in Alternative Healthy Eating Index Score over the 6-Year Exposure Period and Long-term Interleukin-6 Measured Twice over the Subsequent 5-Year Follow-up Period: Supplementary Analyses

Change∗ in AHEI Score over the 6-Year Exposure Period (Between 1991/1993 and 1997/1999)	Participants with IL-6 Measures Available at 1997/1999 or 2002/2004		
	% Difference	SE	*P* Value
Maintaining high score vs maintaining low score			
Model 1	−7.7	2.1	.0001
Model 2	−4.2	2.0	.0311
Improving score vs maintaining low score			
Model 1	−7.0	2.2	.0010
Model 2	−5.1	2.1	.0128
Deteriorating score vs maintaining high score			
Model 1	−4.1	3.0	.1596
Model 2	−4.4	2.8	.1090

Model 1: partially adjusted model: adjusted for sex, age, ethnic group, socioeconomic status, and use of anti-inflammatory drugs. Model 2: fully adjusted model: adjusted as in model 1 + living alone, smoking status, physical activity, total energy intake, hypertension, body mass index, diabetes, coronary heart diseases, and high-density lipoprotein cholesterol. % difference (exp[linear regression coefficient] – 1)*100; a negative percentage difference expressed the percentage reduction in IL-6 levels in the relevant exposure group compared with the reference group. AHEI = Alternative Healthy Eating Index; IL = interleukin; SE = standard error.

∗ To analyze sustained adherence to a healthy diet and improvements in diet, AHEI scores in 1991/1993 and 1997/1999 were categorized as high or low according to the highest quartile of AHEI score in 1991/1993.^32^ Thus, 4 categories of change in AHEI score over a 6-year period were defined: participants who maintained a high score (1991/1993 and 1997/1999 scores ≥59.5, n = 740), participants who maintained a low score (1991/1993 and 1997/1999 scores <59.5, n = 2804), participants whose AHEI score improved (1991/1993 score <59.5 and 1997/1999 score≥59.5, n = 612), and participants whose AHEI score deteriorated (1991/1993 score ≥59.5 and 1997/1999 score <59.5 points, n = 444).

**Appendix Table 3:** Association between Change in Alternative Healthy Eating Index Score over the 6-Year Exposure Period and Long-Term C-reactive Protein Measured Twice over the Subsequent 5-Year Follow-up Period

Change in AHEI Score over the 6-Year Exposure Period (Between 1991/1993 and 1997/1999)	Participants with CRP Measures		
	% Difference	SE	*P* Value
Maintaining high score vs maintaining low score			
Model 1	−17.1	3.3	<.001
Model 2	−12.4	3.1	<.001
Improving score vs maintaining low score			
Model 1	−12.2	4.3	.0023
Model 2	−12.2	3.9	.0010
Deteriorating score vs maintaining high score			
Model 1	18.6	4.5	.0001
Model 2	13.2	4.1	.0027

Model 1: partially adjusted model: adjusted for sex, age, ethnic group, socioeconomic status, and use of anti-inflammatory drugs. Model 2: fully adjusted model: adjusted as in Model 1 + living alone, smoking status, physical activity, total energy intake, hypertension, body mass index, diabetes, coronary heart diseases, and high-density lipoprotein cholesterol. % difference (exp[linear regression coefficient] – 1)*100; a negative percentage difference expressed the percentage reduction in CRP levels in the relevant exposure group compared with the reference group. AHEI = Alternative Healthy Eating Index; CRP = C-reactive protein; SE = standard error.

**Appendix Table 4:** Association between Interleukin-6 Assessed in 1991/1993 and Alternative Healthy Eating Index Score and Its Subsequent Change over the Follow-up

IL-6 Measured in 1991/1993	6-Year Change in AHEI Score between 1991/1993 and 1997/1999				11-Year Change in AHEI Score between 1991/1993 and 2002-2004			
	n	β	SE	*P*	n	β	SE	*P*
Model adjusted for age, sex, ethnicity, SES, and use of anti-inflammatory drugs in 1991/1993	4654	0.066	0.078	.40	3830	0.111	0.096	.25

AHEI = Alternative Healthy Eating Index; IL = interleukin; SE = standard error; SES = socioeconomic status.

**Table 1:** Characteristics in 1991/1993 of Participants Excluded and Included in the Analyses and Mean Long-term Levels of Interleukin-6 Assessed in 1997/1999 and 2002/2004 of Those Included

Variables	Category	N or Mean (SD)∗	Mean Long-term IL-6 (95% CI) (pg/mL) or ρ†	*P* Value‡
Sex	Men	3334	1.65 (1.62-1.68)	.05
	Women	1266	1.59 (1.55-1.64)	
Age	Per year	49.6 (6.1)	0.25	<.001
Ethnicity	White	4317	1.61 (1.59-1.64)	<.001
	South Asian	185	2.07 (1.92-2.23)	
	Black	98	1.63 (1.47-1.81)	
Socioeconomic status	Low	542	1.78 (1.70-1.86)	<.001
	Intermediate	2046	1.68 (1.64-1.72)	
	High	2012	1.55 (1.52-1.59)	
Living alone	No	3588	1.61 (1.58-1.63)	.001
	Yes	1012	1.72 (1.67-1.78)	
Smoking habits	Nonsmokers	2402	1.55 (1.52-1.59)	<.001
	Former smokers	1657	1.64 (1.60-1.68)	
	Current smokers	541	1.98 (1.90-2.07)	
Total energy intake	Per kcal/d	2124	−0.05	.002
Physical activity	Inactive	808	1.76 (1.70-1.83)	<.001
	Moderately active	1313	1.62 (1.57-1.67)	
	Active	2479	1.60 (1.57-1.63)	
General health questionnaire depression cases	No	4040	1.63 (1.61-1.66)	.99
	Yes	560	1.63 (1.5-1.70)	
Coronary heart diseases	No	4487	1.63 (1.60-1.65)	.01
	Yes	113	1.84 (1.67-2.02)	
Body mass index	per kg/m²	25.0	0.32	<.001
Hypertension	No	3803	1.58 (1.55-1.60)	<.001
	Yes	797	1.92 (1.86-2.00)	
Diabetes	No	4487	1.63 (1.60-1.65)	.05
	Yes	113	1.79 (1.63-1.98)	
HDL-cholesterol	per mmol/L	1.43 (0.40)	−0.18	<.001
Use of anti-inflammatory drugs	No	3939	1.59 (1.57-1.62)	<.001
	Yes	661	1.90 (1.83-1.97)	

CI = confidence interval; HDL = high-density lipoprotein; IL = interleukin; SD = standard deviation.

∗ N presented for categoric variables and mean (SD) for continuous variables.

† IL-6 geometric means with its 95% CI (pg/mL) shown for categoric variables and Spearman correlation coefficient for continuous variables.

‡ *P* value for association with chronic IL-6.

**Table 2:** Association between Change in Alternative Healthy Eating Index Score over the 6-Year Exposure Period and Long-term Interleukin-6 Measured Twice over the Subsequent 5-Year Follow-up Period

Change in AHEI Score over the 6-Year Exposure Period (between 1991/1993 and 1997/1999)	Long-term IL-6 Levels					
	In the 4600 Participants with IL-6 Measures Available at 1997/1999 or 2002/2004			In the 3632 Participants with IL-6 Measures Available at 1997/1999 and 2002/2004		
	% Difference	SE	*P* Value	% Difference	SE	*P* Value
Maintaining high score vs maintaining low score						
Model 1	−11.5	1.7	<.001	−11.0	1.8	<.001
Model 2	−8.6	1.6	<.001	−8.2	1.8	<.001
Improving score vs maintaining low score						
Model 1	−9.0	2.3	<.001	−7.7	2.4	<.001
Model 2	−8.1	2.1	<.001	−7.8	2.3	<.001
Deteriorating score vs maintaining high score						
Model 1	2.7	2.4	.25	5.0	2.4	.05
Model 2	0.1	2.3	.95	2.4	2.4	.32

Model 1 (partially adjusted): adjusted for sex, age, ethnic group, socioeconomic status, and use of anti-inflammatory drugs. Model 2 (fully adjusted): adjusted as in model 1 + living alone, smoking status, physical activity, total energy intake, coronary heart diseases, hypertension, diabetes, body mass index, and high-density lipoprotein cholesterol. % difference (exp[linear regression coefficient] – 1)*100; a negative percentage difference expressed the percentage reduction in IL-6 levels in the relevant exposure group compared with the reference group. AHEI = Alternative Healthy Eating Index; IL = interleukin; SE = standard error.

## Appendix

### Supplementary Information on the Procedures of Interleukin-6 Measurement in 1997/1999 and 2002/2004

Interleukin (IL)-6, a marker of systemic inflammation, was measured using a high-sensitivity enzyme-linked immunosorbent assay (R&D Systems, Oxford, UK).^1,2^ At both the 1997/1999 and 2002/2004 follow-up screenings, blood samples were collected between 8 am and 1 pm, stored at −80°C, and not thawed or refrozen during storage. Stored serum samples from both phases were analyzed in the same laboratory. Values below the detection limit (0.08 pg/mL) were assigned a value equal to half the detection limit. To examine short-term biological variation and laboratory error, a repeated sample was taken from a subset of 329 participants at phase 7 (average elapse time 24 days; standard deviation, 11.0). Reliability between samples was assessed with Pearson’s correlation coefficients: *r* = 0.63; intra- and inter-assay coefficients of variation were 7.5% and 8.9%, respectively. The correlation coefficient for IL-6 measures at the 2 follow-up screenings was 0.50. Long-term inflammation was assessed as the average of 2 IL-6 measures in 1997/1999 and 2002/2004 (n = 3632) or by a single measure if only 1 was available (n = 968).
